# *Rowlandius dumitrescoae* species group: new diagnosis, key and description of new cave-dwelling species from Brazil (Schizomida, Hubbardiidae)

**DOI:** 10.3897/zookeys.632.9337

**Published:** 2016-11-16

**Authors:** Alessandro Ponce de Leão Giupponi, Gustavo Silva de Miranda, Osvaldo M. Villarreal

**Affiliations:** 1 Laboratório de Referência Nacional em Vetores das Riquetsioses, LIRN-IOC-FIOCRUZ, Manguinhos, 21040-360, Rio de Janeiro, RJ, Brazil; 2 Center for Macroecology, Evolution and Climate, Natural History Museum of Denmark (Zoological Museum), University of Copenhagen, Universitetsparken 15, Copenhagen, Denmark, 2100; 3 Departamento de Invertebrados, Pós-Graduação em Zoologia, Museu Nacional, Universidade Federal do Rio de Janeiro, Quinta da Boa Vista, São Cristóvão, 20.940-040, Rio de Janeiro, RJ, Brazil

**Keywords:** Diversity, Hubbardiinae, Neotropics, Schizomids, Short-tailed whipscorpion, taxonomy

## Abstract

The *Rowlandius dumitrescoae* species group is reviewed and rediagnosed, and its composition is revised. The group now includes *Rowlandius cousinensis*, *Rowlandius decui*, *Rowlandius dumitrescoae*, *Rowlandius insignis*, *Rowlandius linsduarte*, *Rowlandius monensis*, *Rowlandius peckorum*, *Rowlandius potiguar*, *Rowlandius sul*, *Rowlandius ubajara*, and *Rowlandius pedrosoi* **sp. n.** A new species is described from a cave in northeast Brazil (Santa Quitéria, Ceará). Identification keys and distributional maps are provided for the species of the group. Sixteen species of Schizomida, including five of *Rowlandius*, are currently recognized from Brazil.

## Introduction

*Rowlandius* Reddell & Cokendolpher, 1995, is the most diverse Neotropical genus of Schizomida with 63 described species (Reddell and Cokendolpher 1995; Teruel 2012; Teruel et al. 2012; Delgado-Santa and Armas 2013; Santos et al. 2013). Reddell and Cokendolpher (1995) proposed the genus with a broad concept, using characters that could also fit other genera, and was redefined by Teruel (2004). Reddell and Cokendolpher (1995) assumed *Rowlandius* as monophyletic, but this has never been tested in a phylogenetic analysis; on the contrary, the presence of several variable characters within the genus (e.g., the number of setae on the propeltidium and the shape of the spermathecae) indicates the opposite (Teruel et al. 2012). Within *Rowlandius*, the *biconourus* species group was also proposed as monophyletic, but this hypothesis has not been tested either (Teruel et al. 2012).

The species that compose *Rowlandius* were recognized as a species group long before the genus was erected, when almost all species of Schizomida were placed in *Schizomus* Cook, 1899 (the historical “trash can” of the order). The first attempt to subdivide *Schizomus* into species groups was made by Rowland and Reddell (1979a) who proposed seven; one of them, the *dumitrescoae* group, was divided in three complexes: *dumitrescoae*, *primibiconourus* and *viridis* complex. All *Schizomus* species of these complexes were transferred to *Rowlandius* by Reddell and Cokendolpher (1995). Later, new endeavors to detect and define groups within *Rowlandius* were made by Armas (2002), Teruel (2012) and Teruel et al. (2012), but these included only Cuban species and did not cover all morphological variation within the genus.

Almost 80% of *Rowlandius* species with a known male have striking secondary sexual dimorphism, i.e., the male pedipalp segments are much longer than that of the conspecific females. An interesting case of dimorphism is present in *Rowlandius gracilis* Teruel, 2004 and *Rowlandius potiguar* Santos, Ferreira & Buzzato, 2013, where the same population has both heteromorphic males with long pedipalp articles and homeomorphic males with shorter, female-sized pedipalp articles (Teruel 2004; Teruel et al. 2012; Santos et al. 2013; Oliveira and Ferreira 2014).

*Rowlandius* has an extensive geographic distribution, occurring from Cuba to Brazil. A major radiation of the genus seems to have occurred in the Greater Antilles, where the vast majority of the known species are found (Harvey 2003). In contrast, only five species have been described so far from continental South America (*Rowlandius arduus* Armas, Villarreal & Colmenares-García, 2009, *Rowlandius linsduarte* Santos, Dias, Brescovit & Santos, 2008, *Rowlandius potiguar* Santos, Ferreira & Buzzato, 2013, *Rowlandius sul* Cokendolpher & Reddell, 2000 and *Rowlandius ubajara* Santos, Ferreira & Buzzato, 2013). The genus has been recorded from different biomes, including the Brazilian Amazonia, the Brazilian Atlantic forest, and the Venezuelan cloud forest (Santos et al. 2008; Armas et al. 2009). Recently, some species were discovered inhabiting caves or patches of forest inserted in dry areas of Brazil, the Caatinga (Santos et al. 2008; Santos et al. 2013).

In the present article, a new species of *Rowlandius* is described and illustrated from the state of Ceará, northeast Brazil. Additionally, the *Rowlandius dumitrescoae* group is rediagnosed, an identification key to its species is provided, and the relationships of the new species are discussed.

## Material and methods

The material studied is deposited in Museu Nacional, Universidade Federal de Rio de Janeiro (MNRJ) and FIOCRUZ, Instituto Oswaldo Cruz (CAVAISC). Terminology of pedipalps, legs and spermathecae follows Reddell and Cokendolpher (1995) and Moreno-González et al. (2014); flagellum setation follows terminology of Harvey (1992), modified by Cokendolpher and Reddell (1992), Villarreal et al. (2014), Moreno-González et al. (2014) and Monjaraz-Ruedas et al. (2016); cheliceral setation terminology is based on Lawrence (1969) modified by Villarreal et al. (2016). Description format follows Villarreal et al. (2016). The terms α- and β-males are used here for the two different sizes of heteromorphs. Those with extremely long palp segments are α-heteromorphic males, and those with palp lengths intermediate between those of females and those of α-males are called β-heteromorphic males.

The keys were built based on the material analyzed and the original descriptions (in the case of species with no specimens available for examination). Males are unknown for *Rowlandius sul* and this species was not included in the male key. The preparation and illustrations of the spermathecae follow Villarreal et al. (2016). Dorsal, ventral, and lateral photos were made with a Leica MZ16 microscope attached to a FujiFilm X10 camera. Pictures of live specimens (courtesy of Denis Rafael Pedroso; Fig. 8) were taken with a Canon PowerShot SX130 IS. To generate the SEM images, the specimens were critical point dried and mounted on stubs using an adhesive copper aluminum tape. The mounted stubs were then coated with platinum-palladium and scanned with a JEOL JSM-6390 LV.

Acronyms used:

AMN
 anterior median notch of the chitinized arch 


Dm
 dorso-median setae of abdomen and flagellum 


Dl
 dorso-lateral setae of the abdomen and flagellum 


LL
 lateral lobe of spermathecae 


ML
 median lobe of spermathecae 


Msp
 microsetae patch of the male flagellum 


Vl
 ventro-lateral setae of the abdomen and flagellum 


### Additional material examined

*Rowlandius ubajara* Santos, Ferreira & Buzzato, 2013: Brazil, *Ceará*, Ubajara, Ubajara National Park, 11–14.i.2013, 3°50'24.42"S 40°54'3.96"W, 869m a.s.l., Carlos Frankl Sperber, Thiago Gechel Kloss, Fabiene Maria de Jesus and Gabriel de Oliveira Lobregart *leg.* (1 male, MNRJ 4270).

*Rowlandius potiguar* Santos, Ferreira & Buzzato, 2013: Brazil, *Rio Grande do Norte*, Martins, 6°5'7.87"S 37°55'6.62"W, 319m a.s.l., C. Fukushima and A. Giupponi *leg.* (8 females, MNRJ 4269).

## Taxonomy

### Hubbardiidae Cook, 1899
Hubbardiinae Cook, 1899
*Rowlandius* Reddell & Cokendolpher, 1995

#### *Rowlandius dumistrocae* species group

**Diagnosis.** Male pedipalps of some species sexually dimorphic, with femur and patella extremely elongated, and femur strongly bent proximally (Figs 3D–E, 4A, B). Male flagellum lanceolate (as in *Rowlandius cousinensis* (Rowland & Reddell, 1979), *Rowlandius dumitrescoae* (Rowland & Reddell, 1979), *Rowlandius insignis* (Hansen in Hansen & Sorensen, 1905), *Rowlandius monensis* (Rowland & Reddell, 1979) and *Rowlandius pedrosoi* sp. n.), subquadrate (as in *Rowlandius linsduarte* and *Rowlandius potiguar*) or ovoid (as in *Rowlandius peckorum* (Rowland & Reddell, 1979) and *Rowlandius ubajara*); male flagellum with rounded dorsal projections (with exception of *Rowlandius dumitrescoae*), never surpassing the lateral borders; male flagellum with posterior border surface (between setae *Dl3*) elevated or flat (more rare). Spermathecae with four lobes, lateral pair long with a curved stalk and a terminal enlarged bulb; median lobes short and digitiform or subconical (Figs 7A, B). Chitinized arch very short (relation width/length = 3.7) with acute lateral tips (*Rowlandius cousinensis*, *Rowlandius linsduarteae*, *Rowlandius monensis*, *Rowlandius pedrosoi* sp. n., *Rowlandius potiguar* and *Rowlandius ubajara*) or rounded lateral tip (*Rowlandius dumitrescoae*, *Rowlandius insignis*, *Rowlandius peckorum* and *Rowlandius sul*); anteromedian notch contacting the posterior branch in some species. Gonopod absent. The species included in this group can be checked in Table 4.

**Distribution.** Brazil, Costa Rica, Cuba, Jamaica, Martinique (Windward Islands) and Puerto Rico (Fig. 9).

##### Rowlandius pedrosoi sp. n.

Taxon classificationAnimaliaSchizomidaHubbardiidae

http://zoobank.org/D6088B71-0770-44CD-8283-2CE412AE608C

Figures 1 , 2 , 3 , 4 , 5 , 6 , 7 , 8 , Tables 3 and 4

###### Diagnosis.

Large specimens, male body total length 4.01mm, females 3.85mm (chelicerae and flagellum not included). Spermathecae similar to *Rowlandius potiguar*, but stalk of LL thicker and curved in the apical third; *Rowlandius pedrosoi* sp. n. with stalk of LL and ML with several glandular pores. Lateral tip of chitinized arch “V-shaped”, with obtuse angle, greater than 150°, which distinguishes *Rowlandius pedrosoi* sp. n. from *Rowlandius potiguar* and *Rowlandius linsduarte*. Heteromorphic males present, with α (long pedipalps) and β (shorter pedipalps, but longer than those of females) heteromorphics, similar to *Rowlandius potiguar*. Male flagellum with setae *Dm1* exactly between the main globose area of the flagellum and the stalk, such as in *Rowlandius linsduarte* and differently from *Rowlandius potiguar* and *Rowlandius ubajara*.

###### Type material.

**Holotype**: Brazil, *Ceará*, Santa Quitéria, Gruta P-08, 41529 mE / 9495881 mN SAD‘69S, 15–21.vii.2014, Pellegatti and Pedroso *leg.* (1 male, MNRJ 04266). **Paratypes**: same data as holotype (1 male, 7 females and 10 juveniles, MNRJ 04267); same data as holotype (1 female and 1 juvenile, CAVAISC-ARAC 0008); same data as holotype, 03–13.ii.2014 (4 females and 8 juveniles, MNRJ 04268).

###### Etymology.

The species name is in honor of arachnologist Denis Rafael Pedroso, friend and collector of the type series (of this and many other new species of arachnids).

###### Description.

**Figure 1.**
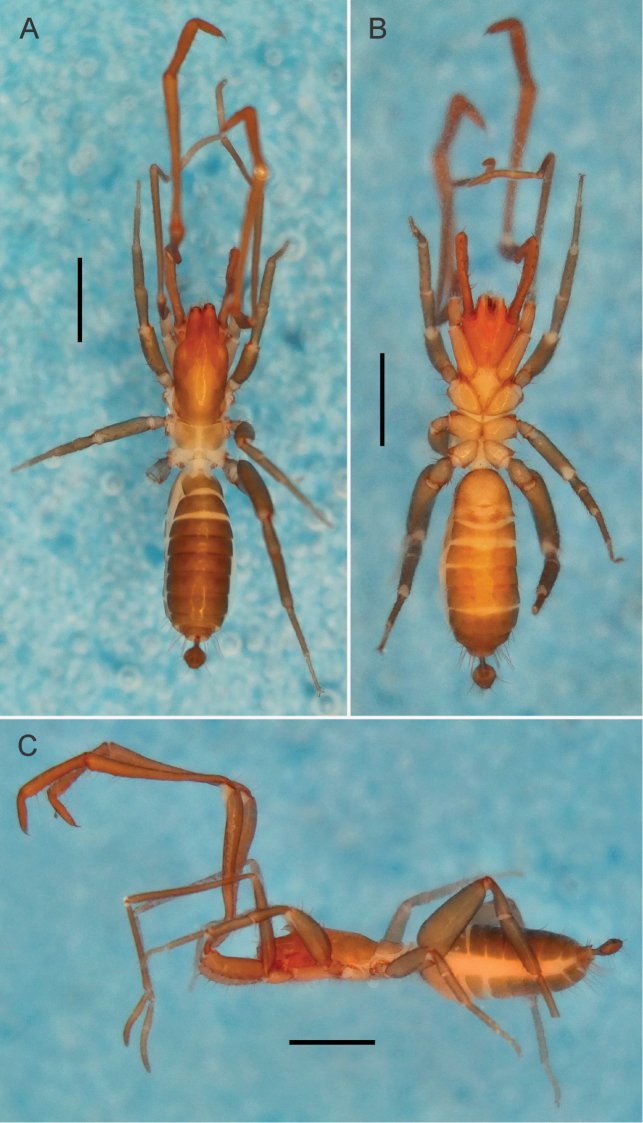
Habitus of an α-heteromorphic male of *Rowlandius pedrosoi* sp. n. (MNRJ 04266). **A** Dorsal view **B** Ventral view **C** Lateral view. Scale bars 1 mm.

*Male holotype*. Color (Fig. 8E–F): live animals with abdominal tergites and sternites olive-brown; pleura white. Pedipalps reddish-brown; legs light brown with the extremities dark-brown. Prosoma light brown; ventral region lighter than the dorsal. Alcohol preserved specimens (Fig. 1) with propeltidium and chelicerae reddish-brown, meso and metapeltidium yellowish-brown (lighter than the chelicerae and propeltidium), legs light brown, abdominal tergites brown and sternites yellowish-brown, flagellum medium-brown. Ventrally coxae I-IV and sternal region yellowish. All body setation light reddish-brown.

**Prosoma** (Fig. 1). Anterior process of propeltidium with two setae (one behind the other) followed by two pairs of dorsosubmedian transversally oriented setae; eyespot suboval; metapeltidium divided. Anterior sternum with 11+2 setae and posterior sternum with 5 setae. Anterior process as wide as long, with a wide base, narrowing abruptly, forming an almost right triangle; the tip of the process is curved downwards.

**Opisthosoma** (Fig. 1). Setae: Tergite I with two pairs of anterior microsetae and one pair of large *Dm* setae. Tergite II with three pairs of anterior microsetae parallel to each other, and one large pair of *Dm* setae. Tergites III–IX and XII each with one pair of large *Dm* setae; VIII with small *Dl2*; IX without *Dm*, but pairs *Dl1* and *Dl2* present; X without dorsal setae; XI with *Dl1* and without *Dl2*; XII with short rounded posterodorsal process and with setae *Dl1* and *Dl2*. Abdominal apodemes with coloration identical to the rest of the sternites. Sternites I–II with many scattered microsetae. Sternite III with 22 microsetae. Sternite IV with *Vl2*, *Vl1* and *Vm2* plus four *AS* microsetae. Sternite V with *Vl2*, *Vl1A*, *Vl1B* and *Vm2*, plus six AS. Sternite VI with *Vm1*, *Vm2*, *Vl1A*, *Vl1B*, *Vl2*, plus six *AS*. Sternite VII with *Vm2*, *Vl1* (A and B), *Vl2*, six *AS* and without *Vm1*. Sternite VIII with *Vm2*, *Vl1*, *Vl2*, plus six *AS*. Sternite IX with *Vm1*, *Vm2*, *Vl1* and *Vl2* plus one pair of supranumeric setae between *Vl1* and *Vm2*. Sternite X with *Vm1*, *Vm2*, *Vl1* and *Vl2*. Sternite XI with *Vm1*, *Vm2* and *Vl1*. Sternite XII with six setae plus four microsetae.

**Figure 2.**
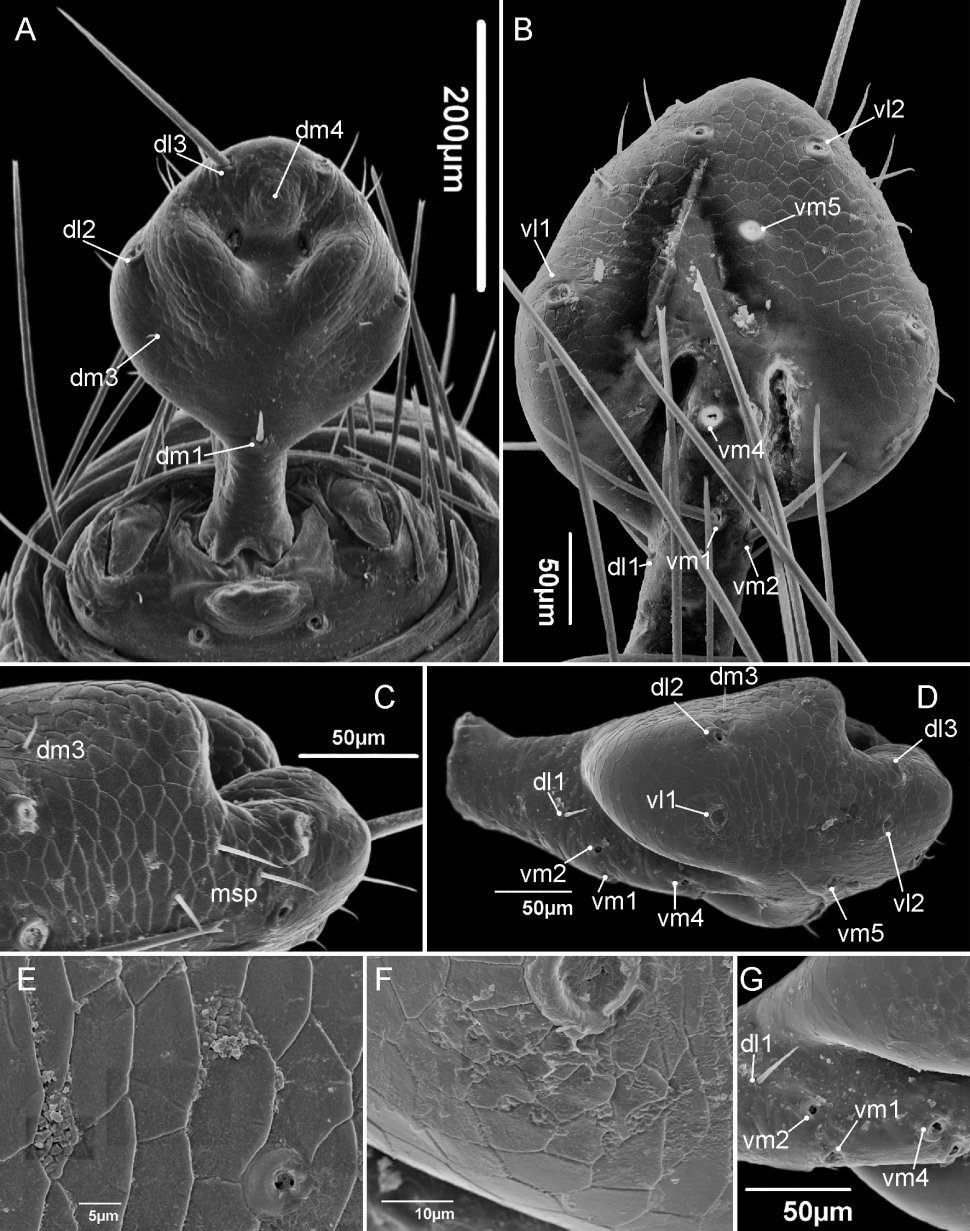
Male flagellum of *Rowlandius pedrosoi* sp. n. (MNRJ 04267). **A** Dorsal view **B** Ventral view **C** Detail in distolateral view **D** Lateral view **E** Uropygi gland opening **F** A set of glands below *VL1* **G** Detail of the position of the proximal ventral and lateral setae.

**Flagellum** (Fig. 2). In dorsal view flagellum diamond shaped, as wide as long, with rounded lateral and apical tips; with three bulges: a pair positioned dorsosubmedian (each bulge seated on opposite sides), without setae, separated by a depression, and one bulge in the central distal region (posteromedian), with the setae *Dm4* on its apex; the central distal bulge is not connected to the lateral ones, with a depression between them. *Dm1* is exactly on the edge between the diamond-shaped part and the stalk. *Dl3* is positioned distally in relation to *Dm4*. Ventrally, *Vm5* is closer to *Vl2* than to *Vl1* and *Vm4*. *Vm1* is closer to *Vm4* than to *Vm2*. Three microsetae on the lateral of the flagellum (msp), between the pairs *Dl2*/*Vl1* and *Dl3*/*Vl2*, closer to the latter. *Dl1*, *Vl1* and *Vl2* forming a straight line in the frontal axis. *Female flagellum* (Fig. 6A–C) with four flagellomeres (I=II=III>IV), wider between the second and third flagellomeres. Dorsally with a small *Dm1* close to the distal margin of the first flagellomere, placed in the middle line; a pair of larger *Dl1* on the wider portion of flagellum, in the point between the second and third flagellomere; one large *Dm4* in the apical portion of the third flagellomere; a pair of small *Dl4* on the fourth flagellomere in mediolateral position; a pair of large *Dl3* apically on the terminal position of the flagellum. Ventrally with a small basal *Vm1* on the first flagellomere, positioned near the distal border; a pair of median *Vm4* in the second flagellomere; one large medial placed *Vm5* on the third flagellomere; a pair of a large *Vl1* on wider portion of the flagellum, between the second and third flagellomeres; a pair of large *Vl2* on the fourth flagellomeres, apically.

**Figure 3.**
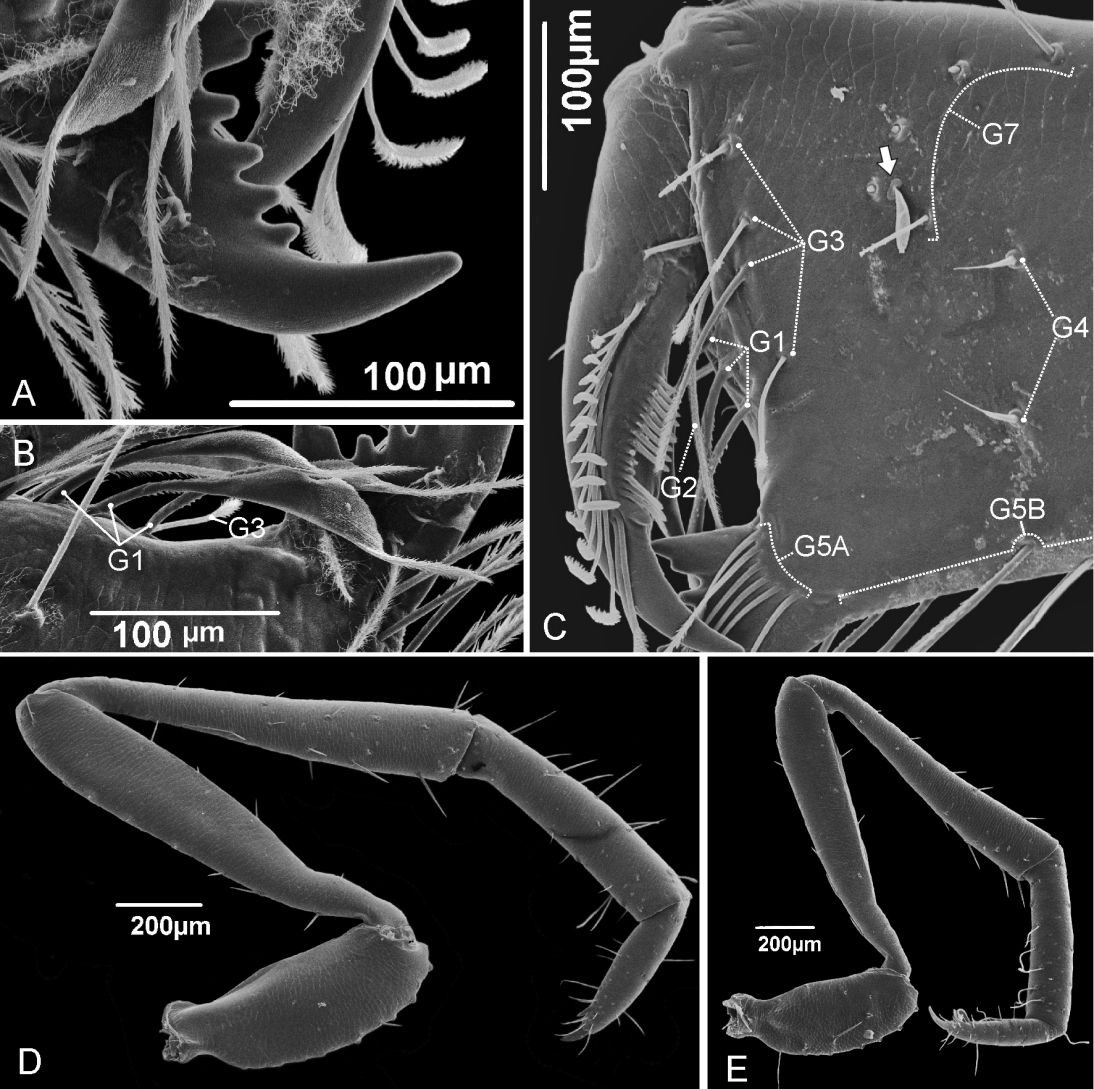
Details of the chelicera and pedipalps of *Rowlandius pedrosoi* sp. n., male (MNRJ 04267). **A** Fixed finger of chelicera **B** Cheliceral setae G1 **C** Mesal view of right chelicera showing setal groups; the arrow indicates the *Basidiobolus* fungus **D** Right pedipalp of a β-heteromorphic, ectal view **E** Left pedipalp of a β-heteromorphic, mesal view.

**Chelicerae** (Fig. 3A–C). Movable finger sharp and curved; serrula with 16 hyaline teeth increasing in size towards distal region; guard tooth rounded. Lamella smooth. Fixed finger with bifid basal tooth, followed by four small subequal teeth; last tooth is the biggest, recurved, with an acute apex, subequal to the basal cusp of bifid tooth. Setation: G1 (setae group 1) with 3 spatulate setae; G2 with 4 feathered setae; G3 with 4 setae, all feathered dorsally and with serrated ventral surfaces; G4 with 2 setae, smooth, short and thick with thin apex; G5A with 6 similar sized feathered setae; G5B with 9 setae larger than G5A; G6 with 1 smooth setae longer than half of movable finger length; G7 with 6 setae decreasing in size from proximal to distal, feathered from the middle to its end. Setal group formula: 3–4–4–2–6–9–1–6.

**Figure 4.**
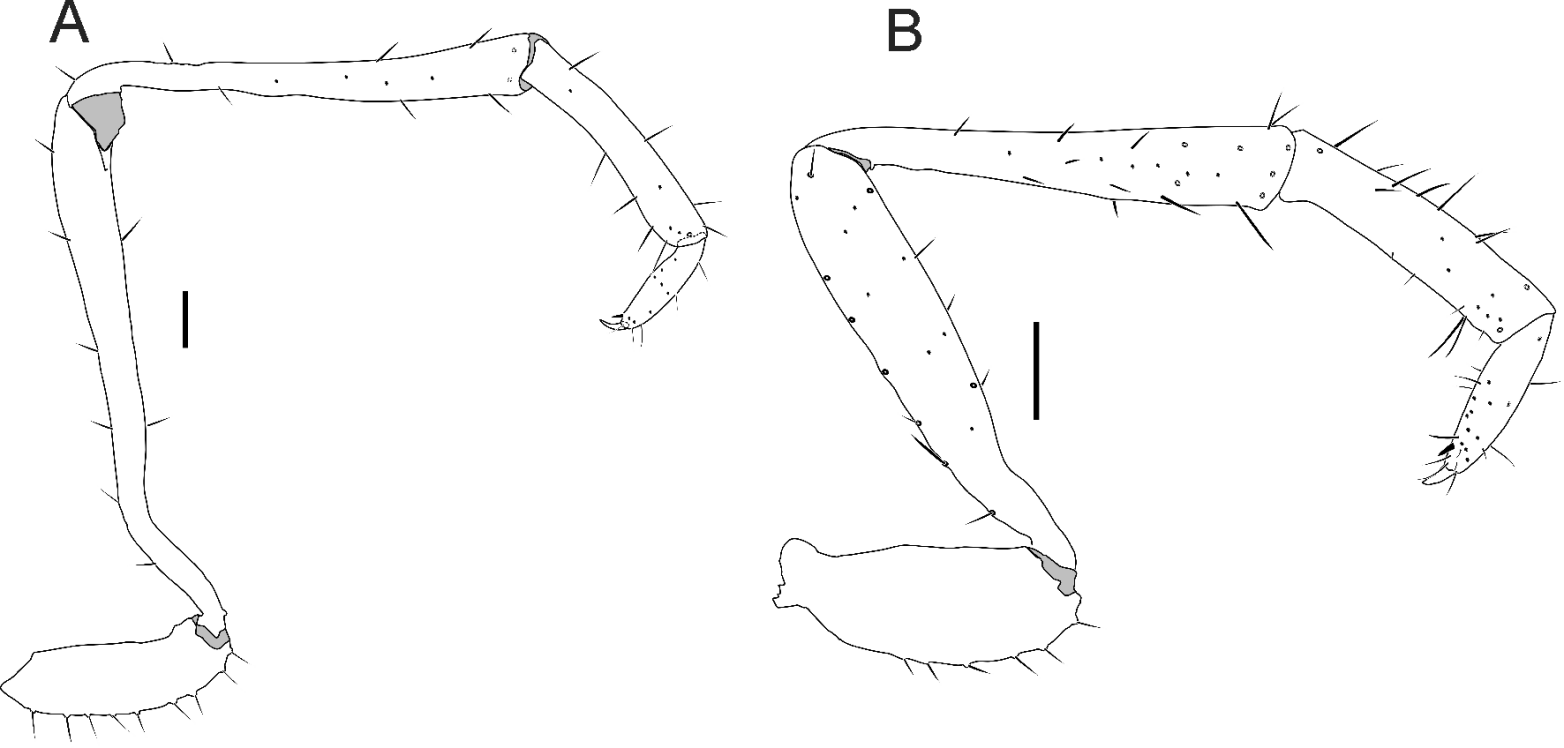
Right pedipalps of heteromorphic males of *Rowlandius pedrosoi* sp. n., ectal view (MNRJ 04267). **A** α-heteromorphic **B** β-heteromorphic. Scale bars 0.2 mm.

**Figure 5.**
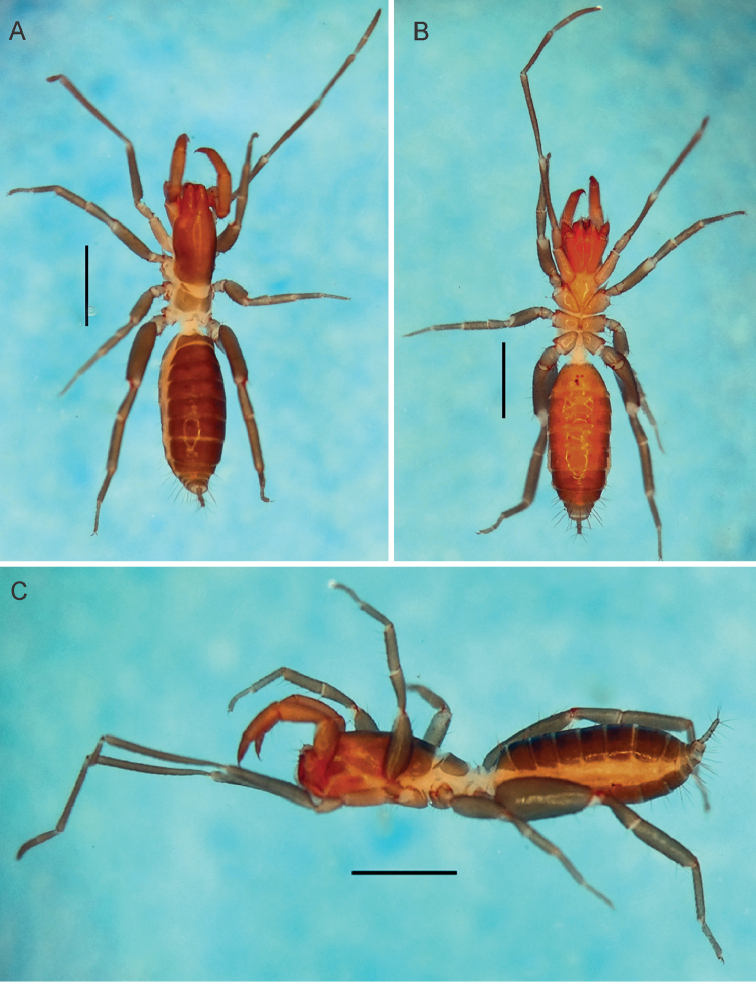
Habitus of a female of *Rowlandius pedrosoi* sp. n. (MNRJ 04267). **A** Dorsal view **B** Ventral view **C** Lateral view. Scale bars 1 mm.

**Pedipalp** (Figs 3D–E, 4). All segments without spinose setae. **Trochanter**: subcylindrical in α-heteromorphic males (in lateral view), longer than wide, with apical portion curved upward; short trapezoid in β-heteromorphic males and even shorter in females (Fig. 5); without apical spur (frontal projection); one ventral row of eight large setae with an intermediate row of three small setae. **Femur**: subcylindrical, club-shaped, with distal portion two times wider than the basal part; in α-heteromorphic males the femur is longer than the total length of the prosoma (pro-, meso- and metapeltidium together); in α-heteromorphic males the femur is longer than the patella (in β-heteromorphic males the femur and patella are subequal and in females the patella is longer); with few setae, only one ventral and one dorsal row of setae; on the ectal surface only one apical setae; on the mesal surface, one row of three setae. **Patella**: subcylindrical, club-shaped, with distal portion two times wider than the basal part; more setae than the femur, with two dorsal and two ventral rows, and four setae on the ectal surface. **Tibia**: cylindrical, α-heteromorphic males with distal portion slightly wider; shorter than half the length of the femur and patella; in β-heteromorphic males and females, the tibia, femur and the patella have similar length. The tibia has the largest number of setae on the pedipalps, with some feather-like setae on the ventral region. **Tarsus**: conical, shorter than the tibia, with lots of setae in the distal third, with two dorsolateral and two ventrolateral rows of setae; two ventrodistal spines pointing forward; tarsal claw sharp and curved, slightly larger than half the tibia length; tarsal spur present.

**Figure 6.**
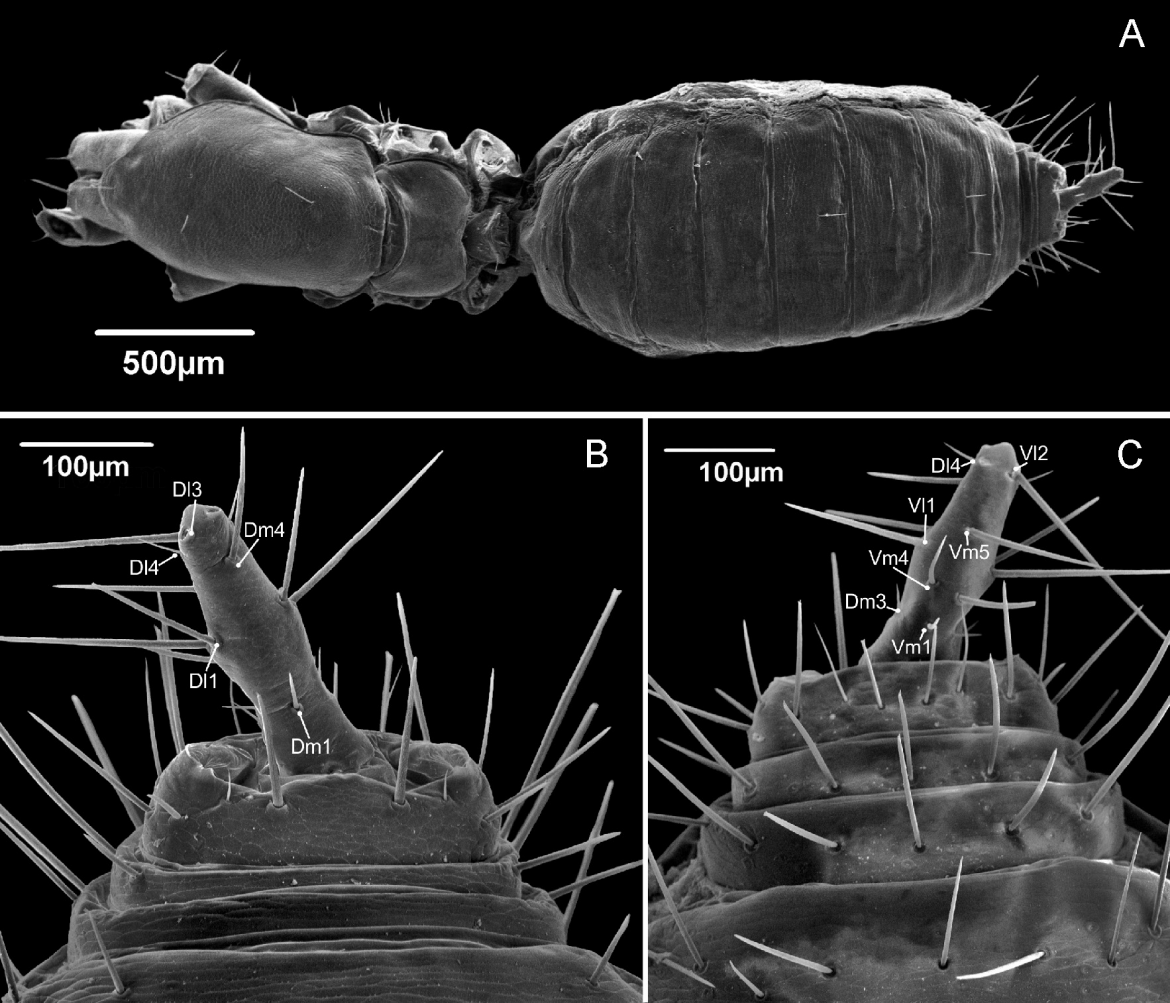
Details of prosoma, opisthosoma and abdomen of a female of *Rowlandius pedrosoi* sp. n. (MNRJ 04267). **A** Dorsal view of prosoma and opisthosoma **B** Dorsal view of female flagellum **C** Ventral view of female flagellum.

**Figure 7.**
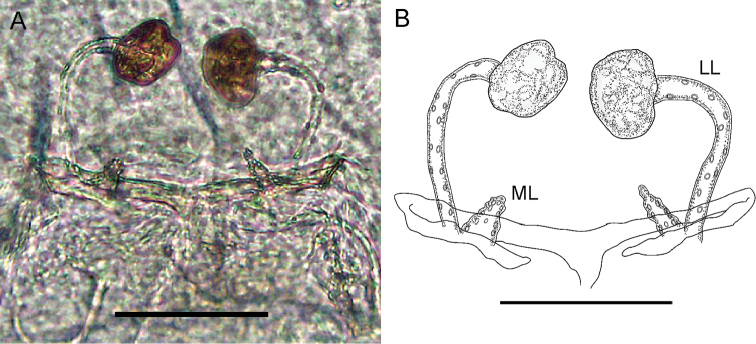
Spermathecae of *Rowlandius pedrosoi* sp. n. (MNRJ 04267). **A** Dorsal view picture **B** Schematic drawing. Scale bars 100 µm.

**Spermathecae of paratype** (Fig. 7). Two pairs of lobes; stalk of the lateral lobe (LL) long, curved (the tips close to each other) and very light colored (almost transparent); with few granules along the structure. Tip of the LL with a wrinkled, rounded structure (resembling a walnut), brown colored (which means it is sclerotized), of about half width of the stalk. The bases of LLs are separated by a distance similar to their lengths. The median lobes (ML) are short, cone-shaped, with a wide base and thin apex; its length is less than a third the size of the LL stalk; the integument is wrinkled with folds on its surface. Bases of the two lobes in contact. The chitinized arch is wider than long, cordiform (or as a “V”, as described by Santos et al. 2013), similar to *Rowlandius potiguar*, however, in *Rowlandius pedrosoi* sp. n. the arch is strongly flattened. In *Rowlandius potiguar*, the vertex of the “V” has about 90–100° (a right angle tending towards the obtuse); in *Rowlandius pedrosoi* sp. n. the same vertex is clearly more obtuse than 150°.

**Figure 8.**
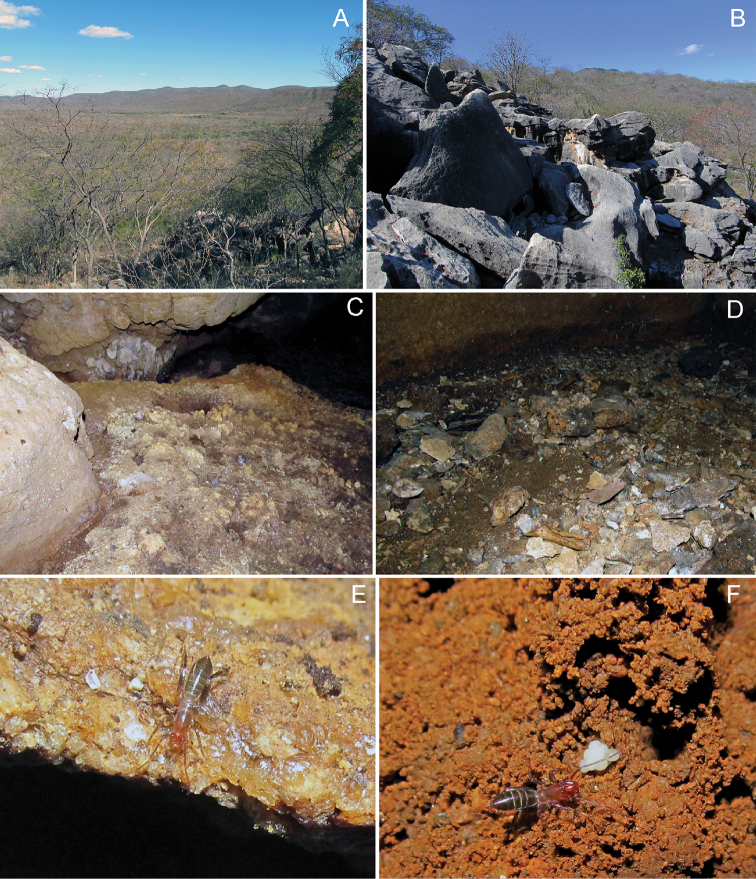
Habitat of *Rowlandius pedrosoi* sp. n. **A** view of the landscape where the cave is located **B** Entrance of the cave **C**–**D** Microhabitat inside the cave where the specimens were collected **E** Female wandering on the cave floor **F** Female walking over some eggs.

###### Distribution

**Figure 9.**
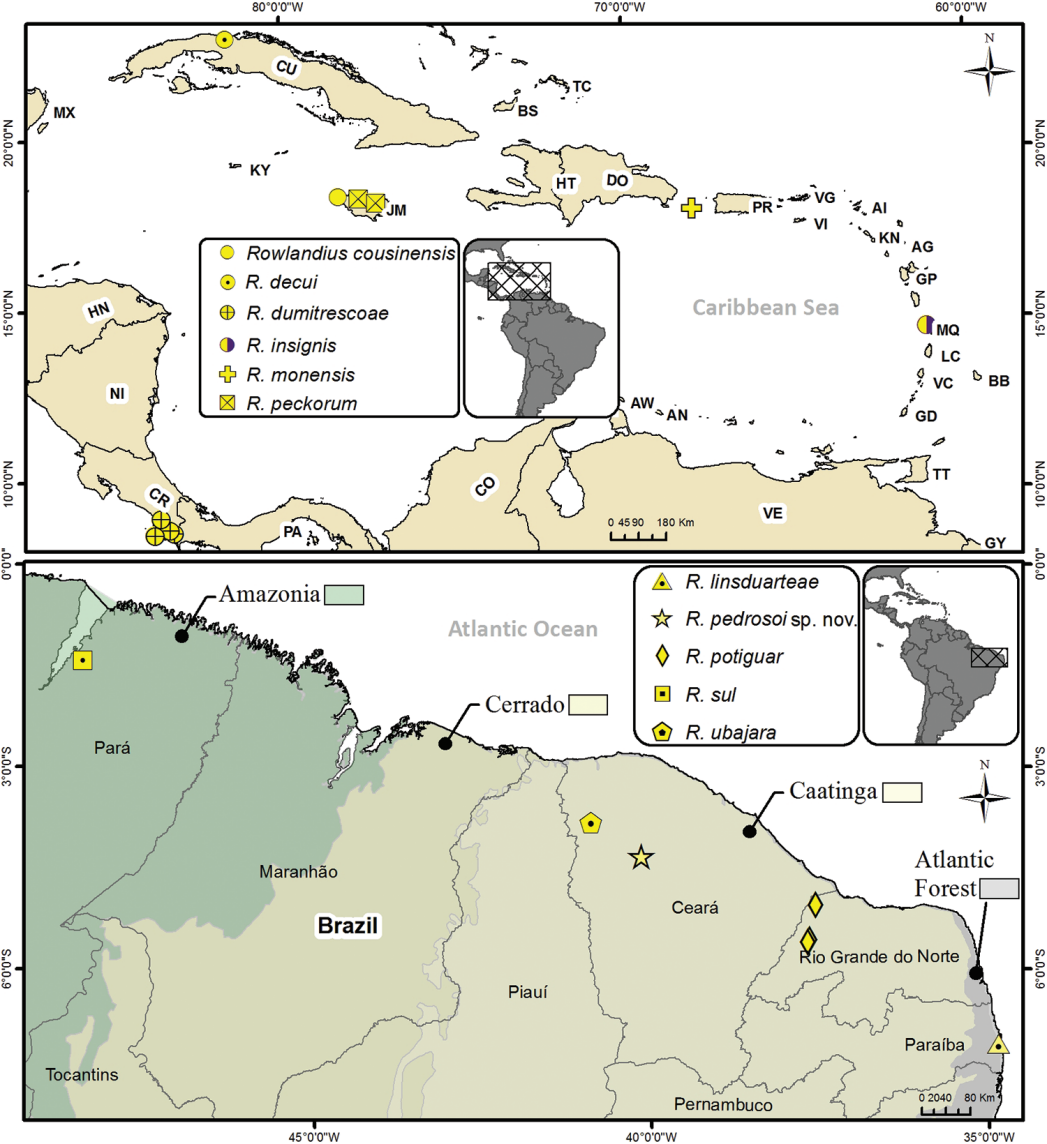
Map showing the distribution of the species of *Rowlandius dumitrescoae* group. The background colors in the Brazilian map represent the biomes.

(Fig. 9). Only known from the type locality: Brazil, Ceará, Santa Quitéria.

###### Natural history.

The type locality is the largest cave in the state of Ceará, formed as a sloping crack (Fig. 8A–B) and with no more than seven square meters of floor space. The specimens were found in one of the few spots with some moisture in the ground. The soil was composed of damp earth of fine sediment agglomerated with gravel, small stones, shells of gastropods and bones from small mammals (Fig. 8C–F). When captured, the schizomids were walking on stones, gravel and debris, where the light barely reached (twilight zone).

Noteworthy of mention is a rare find of a secondary capilliconidium of a (probable) *Basidiobolus* sp. fungus among the cheliceral G7 setae (Fig. 3C, arrow; cf Blackwell and Malloch (1989)). The capilliconidium produces an apical droplet of extracellular material that helps the fungus to attach to and disperse with the host (Dykstra and Bradley-Kerr 1994).

#### Identification keys to the species of the *dumitrescoae* group

##### Key to the males (*Rowlandius sul* male unknown)

**Table:** 

1	Occurs in Brazil	**2**
–	Occurs in the Caribbean or Central America	**5**
2	Male pedipalp trochanter trapezoid in mesal view, with biggest edge facing downwards; apical region of trochanter with a small protrusion that does not touch the articulation of the trochanter-femur; pedipalps showing sexual dimorphism, i.e. larger than those of females; males with heteromorphs; posterodorsal process-XII long	**3**
–	Male pedipalp trochanter cylindrical in mesal view; apical region of trochanter without a small protrusion (all apical region is the articulation trochanter-femur); males without heteromorphs; pedipalps without sexual dimorphism; posterodorsal process-XII short	**4**
3	Posterodorsal process on abdominal segment XII with wide base (exceeding the width of the flagellum pedicel), with rhombus apex, almost as wide as the base of the process; flagellum wider in the basal third; in dorsal view, the dorsal projections of the flagellum reach the lateral border of the flagellum (see Santos et al. 2013, fig. 3A)	***Rowlandius ubajara*** (state of Ceará)
–	Posterodorsal process on abdominal segment XII with narrow base (not exceeding the width of the flagellum pedicel), with thin apex (much narrower than the base); flagellum wider in the median region; in dorsal view the dorsal projections do not reach or surpass the lateral borders of the flagellum (see Santos et al. 2008, fig. 1)	***Rowlandius linsduarte*** (state of Paraíba)
4	Posterodorsal process on abdominal segment XII wider than long; base of the male flagellum dorsal projections not connected, i.e. with a median projection between them (see Santos et al. 2013, fig. 4A, 5A)	***Rowlandius potiguar*** (state of Rio Grande do Norte)
–	Posterodorsal process on abdominal segment XII longer than wide (Fig. 6); base of the flagellum dorsal projections connected, i.e. without the median projection between them (Fig. 2)	***Rowlandius pedrosoi* sp. n.** (state of Ceará)
5	Flagellum with one posteromedian depression	**6**
–	Flagellum without a posteromedian depression	**7**
6	Flagellum with dorsal risings in lateral view	***Rowlandius decui*** (Cuba)
–	Flagellum without dorsal risings in lateral view	***Rowlandius dumitrescoae*** (Costa Rica)
7	Dorsum of flagellum, in lateral view, with big median rising, connected by a parabola-shaped region between lateral and posterior bulge	***Rowlandius cousinensis*** (Jamaica)
–	Dorsum of flagellum, in lateral view, flat-shaped between lateral and posterior bulge	8
8	Pedipalp dimorphic (elongated segments); flagellum in lateral view with median region and stalk at the same level	***Rowlandius insignis*** (Martinique)
–	Pedipalp not dimorphic; flagellum in lateral view with median region higher than the level of the stalk	**9**
9	Flagellum lanceolate; flagellum in lateral view with flat posterior region	***Rowlandius monensis*** (Jamaica)
–	Flagellum nearly globose; flagellum in lateral view with elevated posterior region	***Rowlandius peckorum*** (Puerto Rico)

##### Key to the females

**Table:** 

1	Occurs in Brazil	**2**
–	Occurs in the Caribbean or Central America	**6**
2	Median lobes of spermathecae long, finger shaped; stalk of lateral lobes slightly curved and without globose structure in the apex (slightly wider than the rest of the stalk); chitinized arch procurved	***Rowlandius ubajara*** (state of Ceará)
–	Median lobes of spermathecae short , cone shaped; stalk of lateral lobes curved and with globose structure in the apex; chitinized arch cordiform	**3**
3	Chitinized arch of spermathecae with rounded inferior part (posterior branch); median lobes closer to the anterior part of the chitinized arc	**4**
–	Chitinized arc of spermathecae with “V” shaped inferior part (posterior branch); median lobes closer to the posterior part of the chitinized arc	**5**
4	Lateral lobes of spermathecae with a winding stalk and a small globose structure at the apex (globe less than twice the width of the base)	***Rowlandius sul*** (state of Pará)
–	Lateral lobes of spermathecae with an arched stalk and a large globose structure at the apex (globe twice as wide as the base)	***Rowlandius linsduarte*** (state of Paraíba)
5	Chitinized arch of spermathecae with central region of the anterior part “V” shaped	***Rowlandius potiguar*** (state of Rio Grande do Norte)
–	Chitinized arch of spermathecae with central region of the anterior part almost straight	***Rowlandius pedrosoi* sp. n.** (state of Ceará)
6	Median lobes of spermathecae close to the base of the chitinized arch and distant to the base of the lateral lobes; lateral lobes long, stalk curved, apex discoid	***Rowlandius dumitrescoae*** (Costa Rica)
–	Median lobes of spermathecae distant to the base of the chitinized arch and close to the base of the lateral lobes; lateral lobes long or short, stalk curved or not, and apex rounded or discoid	**7**
7	Lateral lobes of spermathecae short; median and lateral lobes with their bases in the same line (one is not anterior or posterior to the other)	***Rowlandius monensis*** (Puerto Rico)
–	Lateral lobes of spermathecae long; base of the lateral and median lobes not in the same line	**8**
8	Posterior region of the chitinized arch of spermathecae straight	***Rowlandius cousinensis*** (Jamaica)
–	Posterior region of the chitinized arch of spermathecae curved	**9**
9	Lateral and median lobes of spermathecae close to the anterior region of the chitinized arch; median and lateral lobes with their bases in the same line	***Rowlandius peckorum*** (Jamaica)
–	Lateral and median lobes of spermathecae in the center of the chitinized arch; median lobes positioned anteriorly to lateral lobes	***Rowlandius insignis*** (Martinique)

## Discussion

In general, species groups facilitate comparisons and identifications in speciose genera as they comprise a subset of a genus, and make the process of understanding relationships more comprehensible (Passos et al. 2015). Initially, only few genera were recognized in Schizomida and some of these (e.g., *Schizomus* Cook, 1899; *Trithyreus* Kraepelin, 1899) accumulated a number of species, but eventually they were subdivided, first into species groups, some of which were later recognized as new genera (Rowland and Reddell 1979a, b, 1980, 1981). The *dumitrescoae* group is an example of species group that was raised to genus. The group was defined by Rowland and Reddell (1979a) and was later transferred to *Rowlandius* by Reddell and Cokendolpher (1995). At that time, all species were from Central America (see Table 1 and Fig. 9) and were defined by the large body size, carapace with two to four pairs of dorsal and one pair of apical setae, female flagellum with four flagellomeres, spermathecae elongated laterally and reduced in the middle, and a few other characters (Rowland and Reddell 1979a). Afterwards, Armas (2002) proposed other species groups based on Cuban species and defined them using mainly characters of the pedipalp and the spermathecae (Table 2).

**Table 1.** Species groups and complexes proposed by Rowland and Reddell (1979a) and Reddell and Cokendolpher (1995) to the *dumitrescoae* group (when the species were still in *Schizomus* (R&R79) and after being transferred to *Rowlandius* (R&C95)).

Group	Complex	Species
*dumitrescoae* group	*dumitrescoae* complex	*Rowlandius dumitrescoae*
		*Rowlandius decui*
	*primibiconourus* complex	*Rowlandius cousinensis*
		*Rowlandius primibiconourus*
		*Rowlandius longipalpus*
		*Rowlandius brevipatellatus*
	*viridis* complex	*Rowlandius gladiger*
		*Rowlandius monensis*
		*Rowlandius desecho*
		*Rowlandius biconourus*
		*Rowlandius insignis*
		*Rowlandius peckorum*
		*Rowlandius viridis*

**Table 2.** *Rowlandius* species groups and complexes proposed by Armas (2002).

Groups	Subgroups	Species	Diagnostic character
I		*Rowlandius biconourus*	“Presence of a dorsal spur on the heteromorphic pedipalp trochanter of the male.”
		*Rowlandius ramosi*	
		*Rowlandius recuerdo*	
II		*Rowlandius abeli*	“Spermathecae differs significantly from the general pattern present in congeners.”
III		*Rowlandius decui*	“Spermathecae with the terminal bulb underdeveloped and short middle lobe.”
		*Rowlandius digitiger*	
IV		*Rowlandius cubanacan*	“Long and subequal spermathecae with the terminal bulb underdeveloped.”
		*Rowlandius labarcae*	
V	V-1	*Rowlandius negreai*	“Spermathecae with terminal bulbs well developed, with lateral lobes clearly longer and with a larger bulb.”
		*Rowlandius monticola*	
	V-2	*Rowlandius baracoae*	
	V-3	*Rowlandius toldo*	
		*Rowlandius gladiger*	
		*Rowlandius alayoni*	
		*Rowlandius siboney*	
		*Rowlandius terueli*	

**Table 3.** Measurements of *Rowlandius pedrosoi* sp. n. specimens.

Body	Male holotype MNRJ 4266	Female paratype MNRJ 4267
Total body: L	4.01	3.85
Propeltidium: L	1.25	0.98
Propeltidium: W	0.67	0.61
Metapeltidium: L	0.62	0.24
Metapeltidium: W	0.25	0.29
Abdomen: L	2.3	2.00
Abdomen: W	0.9	0.92
Flagellum: L	0.37	0.25
Flagellum: W	0.23	0.07
**Pedipalp: L**		
trochanter	0.82	0.58
femur	2.06	0.56
patella	1.84	0.62
tibia	0.91	0.53
tarsus + claw	0.54	0.41
**Leg: I L**		
coxa	0.42	0.61
trochanter	0.33	0.33
femur	1.18	1.23
patella	1.55	1.53
tibia	1.07	0.99
basitarsus	0.33	0.21
telotarsus	0.55	0.3
**Leg: IV L**		
femur	1.06	1.24
patella	0.51	0.55
tibia	0.82	0.89
basitarsus	0.67	0.67
telotarsus	0.47	0.46

Studies on South American Schizomida revealed *Rowlandius* species inhabiting Brazil (Cokendolpher and Reddell 2000; Santos et al. 2008; Santos et al. 2013) and those species have a set of characters shared with some Caribbean (*Rowlandius cousinensis*, *Rowlandius decui*, *Rowlandius insignis*, *Rowlandius monensis* and *Rowlandius peckorum*) and Central American species (*Rowlandius dumitrescoae*), suggesting that the Brazilian *Rowlandius* fauna also belong to the *dumitrescoae* group. The characters present in all these species are: **1)** female spermathecae with long lateral lobes and with a broad distal expansion, **2)** median lobes short, digitiform without distal expansion, **3)** gonopod absent, **4)** chitinized arch with opened anterior branch (without AMN) and posterior branch rounded (*Rowlandius cousinensis*, *Rowlandius dumitrescoae*, *Rowlandius insignis*, *Rowlandius monensis* and *Rowlandius peckorum*), or anterior branch closed and posterior branch retrocurved (*Rowlandius pedrosoi* sp. n. and *Rowlandius potiguar*) or rounded (*Rowlandius linsduarte*, *Rowlandius ubajara* and *Rowlandius sul*), **5)** males with pedipalp elongated (such as *Rowlandius decui*, *Rowlandius dumitrescoae*, *Rowlandius insignis*, *Rowlandius potiguar* and *Rowlandius pedrosoi* sp. n.), and **6)** male flagellum never trilobate in dorsal view, but diamond-shaped and with dorsal projection (absent in *Rowlandius dumitrescoae* and reduced in *Rowlandius decui*). Santos et al. (2008) already noted that *Rowlandius linsduarte* and *Rowlandius sul* are more closely related to each other than to any other species based on the female genitalia, but did not include them in any group. Here a new composition of the *dumitrescoae* group is proposed based on the above-mentioned characters (see also Table 4).

**Table 4.** List of species maintained, removed, and added to the *dumitrescoae* group.

Species maintained	Species removed	Species added
*Rowlandius cousinensis*	Rowlandius primibiconourus*	*Rowlandius linsduarte*
*Rowlandius decui*	*Rowlandius longipalpus*	*Rowlandius potiguar*
*Rowlandius dumitrescoae*	*Rowlandius gladiger*	*Rowlandius sul*
*Rowlandius monensis*	*Rowlandius desecho*	*Rowlandius ubajara*
*Rowlandius peckorum*	*Rowlandius biconourus*	*Rowlandius pedrosoi* sp. n.
*Rowlandius insignis*	*Rowlandius viridis*	

* This species was removed because its documentation in the literature is insufficient and we had no access to specimen; see discussion for details.

Some *Rowlandius* illustrated in the literature are potentially part of the *dumitrescoae* group, but are not presently included, once no material was accessible during the preparation of the work. One of them is an undescribed species from Tortuguero, Costa Rica, illustrated by Armas (2009) (see fig. 3D); the spermathecae of the specimen fits the present definition of the *dumitrescoae* group, but as the species was not formally described and the male is not known, the correct relationship of the morphospecies cannot be assured by now. Another species that can potentially be part of the group is *Rowlandius viridis*; Rowland and Reddell (1979a) illustrated this species from four localities, and one of them (from Pedro Great Cave, Clarindon Parish) is similar to the standard shape of the *dumitrescoae* group, but since there is a huge variation in the size and shape of the lobes in this species, further studies are needed before reaching a conclusion on those populations.

An interesting character observed in some species of *Rowlandius* (e.g. *Rowlandius dumitrescoae*, *Rowlandius insignis*, *Rowlandius potiguar* and *Rowlandius pedrosoi* sp. n.) is the strong sexual dimorphism of the palps. The femur and patella of the pedipalps are extremely long in α-heteromorphic males compared to females and homeomorphic males, as reported by Santos et al. (2013). Other cases of elongated male-dimorphic appendages in arachnids are found in harvestmen (Orrico and Kury 2009; Buzatto et al. 2011; Zatz et al. 2011) and whip spiders (Vasconcelos et al. 2014). It is possible that the elongate pedipalps of *Rowlandius pedrosoi* sp. n. evolved due to sexual selection pressures, similarly to that found in *Rowlandius potiguar* (Santos et al. 2013).

*Rowlandius* is the only short-tailed whip scorpion genus found in the dry biome of Caatinga (Santos et al. 2008; Santos et al. 2013). The four schizomid species found in that harsh environment (*Rowlandius linsduarte*, *Rowlandius pedrosoi* sp. n., *Rowlandius potiguar* and *Rowlandius ubajara*) are restricted to protected places, such as forests or caves, where the temperature is mild, the humidity is high and the variation these environmental conditions is lower. These species appear to be limited to these hypogean habitats, but they do not have apparent troglomorphisms and their presence in caves may be a recent invasion after climate change in Northeastern Brazil and retraction of the forest (Santos et al. 2007). The small size and the relatively thin cuticle of schizomids makes them sensitive to dehydration and caves serve as a suitable habitat for these animals (Oliveira and Ferreira 2014). The exotic species *Stenochrus portoricensis* Chamberlim, 1922, for example, has already been found in caves in Central Brazil (Gallão et al. 2015).

## Supplementary Material

XML Treatment for Rowlandius pedrosoi
